# Assessment of the psychomotor fitness level of cadet pilots after a 6-month flight training period - pilot study

**DOI:** 10.3389/fpsyg.2023.1205412

**Published:** 2023-08-30

**Authors:** Zbigniew Wochyński, Justyna Skrzyńska-Rękawek, Zdzisław Kobos

**Affiliations:** ^1^Department of Air Transport Safety, Polish Air Force University, Deblin, Poland; ^2^Air force Institute of Aviation, Warsaw, Poland; ^3^Department of Psychology of Work and Stress, Cardinal Stefan Wyszynski University in Warsaw, Warsaw, Poland

**Keywords:** diagnostic and training device, rotation, heart rate, blood pressure, mental condition, sensory system, motor system, synthetic memory (working)

## Abstract

**Objectives:**

The aim of the study was to investigate the impact of 6-month aviation practice on the improvement of physical fitness and cognitive abilities of cadet pilots.

**Materials and methods:**

Twenty cadet pilots (men) with an average age of 22 from the Polish Air Force Academy were tested. Two tests of effort were conducted: before (test I) and after (test II) practical flights. Test of physical effort relyed on the number of rotations of looping accomplished. When performing rotations on looping, in tests I and II, the percentage level of task performance was assessed using a diagnostic and training device. Tasks for the exerciser were sent wirelessly to the glasses from the operator’s position. The heart rate (HR) and blood pressure were measured with a camera-type Microlife AG.

**Results:**

In test II, there was a statistically significant improvement in the percentage of task completion (*p* < 0.01) and an insignificant improvement in the performance of rotations on looping compared to test I. After Test I and II demonstrated a statistically significant increase in HR and systolic blood pressure when compared to before test values. Test II illustrated a significant decrease in systolic blood pressure after effort (*p* < 0.05) with regard to test I.

**Conclusion:**

The study showed that cadet aviation practice ameliorated their physical fitness and cognitive skills, as well as helped to diminish their situational stress, which resulted in a decrease in systolic blood pressure after test II.

## Introduction

A high level of psychomotor fitness in pilots is the basis for performing combat tasks (Tomczak et al., 2017; Wochyński, 2021; Prokopczyk and Wochyński, 2022a,b). This issue does not lose relevance with the technological progression of military aircraft. The pilot’s psychomotor development is the key to quick decision-making, ordering mental processes, and perfect coordination of motor and mental activities (Tomczak, 2015; Tomczak et al., 2019; Wochyński et al., 2020a; Wochyński, 2021, 2022). At the same time, the level of physical fitness and cognitive features of a pilot cannot be treated as separate phenomena. A modern pilot must combine them comprehensively, contributing to solving professional tasks in extreme conditions (Tomczak, 2015; Tomczak et al., 2017). From this point of view, psychomotor tests for military pilots must be mandatory and performed comprehensively. The diagnostics of skills and cognitive processes in pilots is an essential factor that conditions the safety and efficiency of flight missions. This is why, in military aviation, psychological (cognitive, space orientation, and stress resilience) and motor skills (sight-movement coordination and balance) tests were introduced. Advancements in technology forced researchers to seek innovative methods for the assessment of psychomotor skills in the pilot’s training (Prokopczyk and Wochyński, 2022a,b). These methods have to be adequate to the opportunities created by modern aviation technology, for example with regard to aircraft maneuverability, G-forces, armaments, navigation, flight-speed, etc. The traditional procedures of candidate selection include psychological and physical tests which are separate and do not coincide. These separate procedures to assess one’s suitability for a career in aviation negatively influence the diagnostic accuracy of the psychomotor predispositions necessary to perform complicated tasks in the air. The conducted research, reported evaluation, and performed training enable an increase in the diagnostic accuracy of the suitability assessment of the candidates for military aviation training. One of the specialized methods to prepare pilots physically for flight in extreme conditions is training on Special Aerial Gymnastics Instruments (SAGIs; Wochyński et al., 2010). These Instruments are as follows: looping, gyroscope, and aero wheel. Their structure and appearance were presented in the previous papers (Wochyński et al., 2010; Wochyński and Sobiech, 2014, 2015, 2017). The exercises on those instruments partly imitate the real flight mission’s environment. Thus, SAGIs help to evaluate the predispositions needed to perform the mission in real time and the capability to utilize the technological and maneuvering abilities of the aircraft. The level of physical and mental fitness depends on the adaptation and integration processes of the motor and sensory systems. In the time criterion imposed on the conduction of the above-mentioned tasks, physical and mental fitness might be a reason for feedback between them. This is why a new method was implemented, a stress test on looping connected with a diagnostic-training device used to evaluate the cognitive processes (including the ability to concentrate and focus attention, to think) and to evaluate the skills (the accuracy of the accomplishment of assigned tasks).

In this article, a hypothesis was made that a half-year-long practical flight training will have an influence on the amelioration of physical fitness and cognitive skills among cadets. At the same time, it was assumed that the use of the diagnostic-training device will have a high diagnostic value and will allow us to assess the maximum psychomotor threshold, at which feedback will appear (decreasing correlation) between the percent of accomplished tasks and the number of performed rotations after a half-year-long practical flight training.

## Materials and methods

### Participants

Twenty cadet pilots (all men) studying at the Polish Air Force Academy in Dęblin, aged 22 on average, underwent examinations before (test I) and after (test II) a half-year of practical flying training (Table 1). Cadets were a homogeneous material selected by the Military Aeromedical Board, which obtained the highest health category (Z-1A). Looping exercises require special motor adaptation from the participants. Before starting their flight practice, the cadets completed a 40-h program of special exercises on SAGI, preparing for flights within 70 days (Wochyński et al., 2010). Due to the technical difficulty of rotating the looping in the forward direction, the authors did not implement a control group for the study. The criterion for inclusion in the study was: the consent of the subject, exclusion from the study: resignation of the subject, injury, or professional duties.

**Table 1:** Age and somatic data (*n* = 20).

parameter	Test I M ± SD	95% CI	Test II M ± SD	95% CI	*p*
Age (years)	22.0 ± 1.52	21.2; 22.7	22,0 ± 1.52	21.2;22.7	ns
Body weight (kg)	73.4 ± 4.26	70.4; 76.3	74.0 ± 5.83	71.2;76.7	ns
Body height (cm)	177.8 ± 4.38	175.6;179.8	177.8 ± 4.38	175.7;179.7	ns
BMI (kg.mˉ^2^)	23.3 ± 1.49	22.5; 23.9	23.4 ± 1.57	22.6;24.1	ns

M ± SD- mean, standard deviation; CI- confidence interval; *p*-level of significance; ns-statistical insignificance.

### Examinations

Cadets’ heart rate (HR), systolic, and diastolic blood pressure were measured with an electronic device of the Microlife AG type before and after test I and II.

## Method

During the examinations, a stress test on a looping, which is one of the SAGI devices, was used (ability to perform exercises, motor coordination), combined with the application of a diagnostic and training device developed to assess mental agility (the ability to concentrate and focus) as well as abilities and skills (precision of performing the task). Each of the tested cadets was wearing the diagnostic and training device. Looping is an ongoing training tool for pilots and has also been used to develop the psychomotor test (Prokopczyk and Wochyński, 2022a,b) and the assessment of vegetative symptoms (Wochyński et al. 2020a,b). Rotational looping exercises cause overloads of +Gz and-Gz in the subjects, therefore the cognitive processes of cadet pilots were assessed in conditions close to real.

### Description the device

The device consists of a backpack with a small computer controlling the cognitive tasks displayed in the goggles. The experimenter’s workstation allowed for online monitoring of the reactions of the tested person in whom tasks appeared in the goggles. The task simulation was applied wirelessly from the main workstation to the trainee via a relay station.

### Description of the test

**Figure 1:**
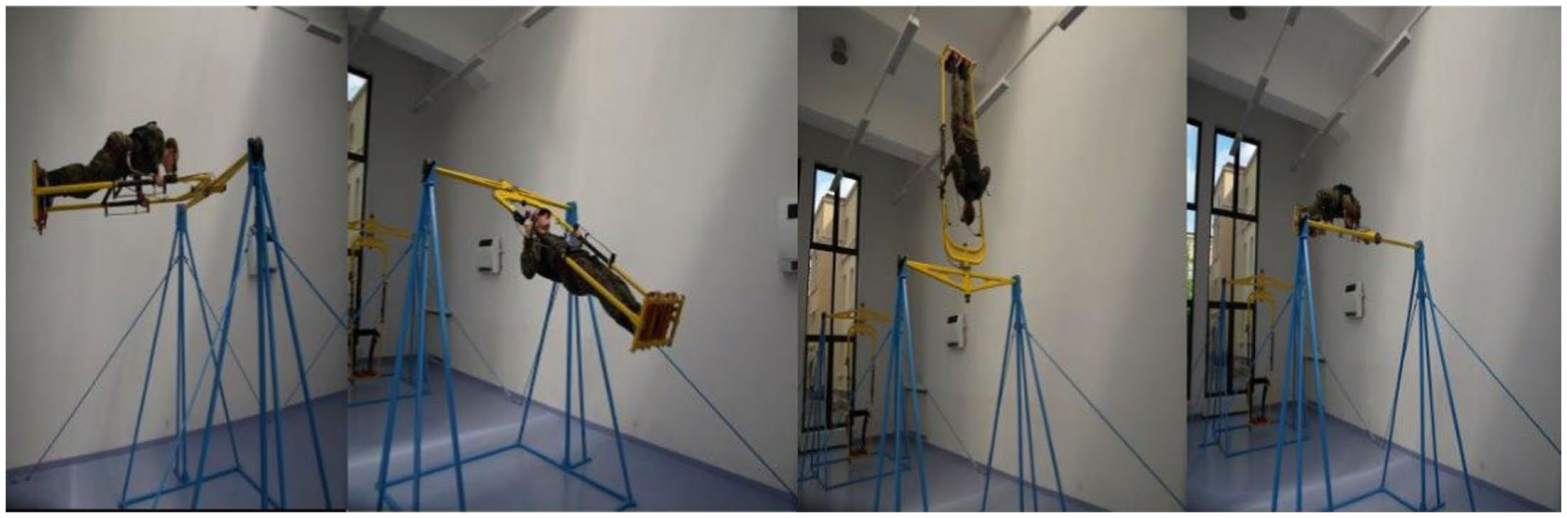
The sequence of forword rotation on a looping with a diagnostic and training device.

After initiating the looping, when their body was moving in the tridimensional space, the cadets were assigned 5 cognitive tasks to complete within the timeframe of 128 s, which were displayed in their goggles. The following were established for each of the tasks: the time cadets waited for a question (5 s), the time the question was displayed (10 s), and the length of the sequence (10 s). The efficiency of the accomplishment of the cognitive tasks was assessed in percentage. Whereas physical fitness was assessed with the number of performed loops (Figure 1). It was assumed that all the tasks were performed correctly. After performing the looping exercises the tested person moved on to performing the sixth task – a synthetic memory test. The synthetic memory test consisted of listing the order of performing the tasks during the exercises.

The type of tasks displayed in the goggles and their evaluation was as follows:

1. To count parachutists in one color - correct
2. To count cars in one color - correct
3. To count shapes of one type - correct
4. Arithmetic operations - correct
5. To count shapes in one color – correct
  Grade: 100%.

The task simulation program had the function of changing the order of their display in the study.

After completing the looping test, the cadets underwent a memory test:

1. Synthetic memory test – correct
  Grade: 100%.

### Flying practice program

During their flying practice, the cadets were performing certain types of program flights in the air. Moreover, they were participating in physical education classes. The classes were very intensive to increase the pilot’s physical fitness. During the training tasks, repetitive and interval method was applied. The cadets followed the physical education program two times a week, with 2 h each session, while in the scope of directed physical training they also had 2-h-long sports classes once a week. During the implementation of the training program, all subjects were provided with the same accommodation and food conditions.

### Statistical analysis

The average and standard deviation of all the variables were counted in a statistical study. A normal distribution of all the examined variables was checked by means of the Kolmogorov–Smirnov test. In order to verify the homogeneity of variance, Levene’s test was applied. The difference in results between tests I and II was calculated by analysis of variance (ANOVA) with repeated measurements using the Tukey HSD posthoc test for pairwise comparison. In tests I and II, the r-Pearson’s correlation between the task completion percentage and the number of rotations while looping was calculated. In the statistical study of all variables, the value of the effect size was calculated using Cohen’s d test. The following criteria were used to evaluate Cohen’s d-test: 0.2 to 0.3 small, 0.5 medium, and greater than 0.8 large effect size (Cohen, 1988). The statistical analysis of the test results was performed using the STATISTICA 13.3 statistical program, the G* power program was used to assess the sample size (Faul et al., 2007). To evaluate the sample size with the size effect f^2^ = 0.25 was assumed an alfa error of 0.05 and a test‘s power of 0.80. The required size of the total sample was estimated at 25 people. Due to professional duties, the final analysis included 20 people. The differences in averages are considered significant when the calculated value of *p* is smaller than 0.05.

## Results

In test II, in comparison to test I, no significant changes in somatic parameters were observed (Table 1). Test I and II demonstrated a statistically significant increase in HR and systolic blood pressure after the stress test when compared to the values before it.

**Figure 2:**
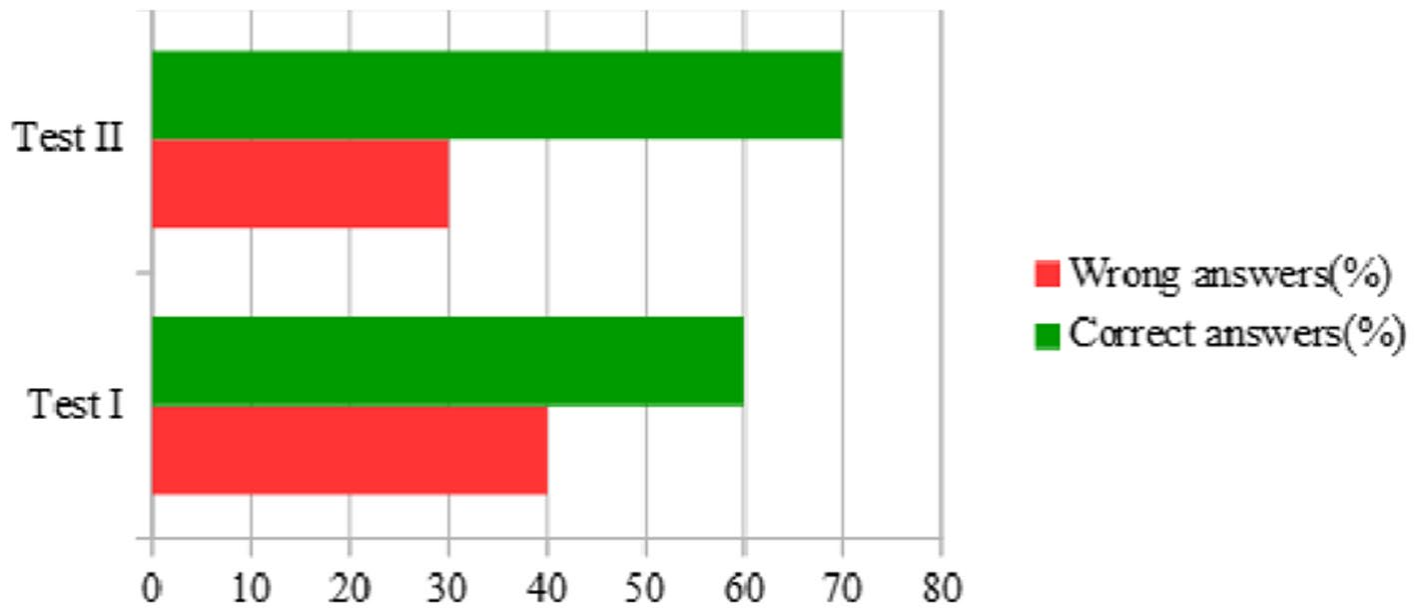
Percentage distribution result of the synthetic memory test I and II (*n* = 20).

In test I after the exercise a statistically significant increase in blood systolic pressure was observed in comparison to the value before the exercise. An increase in HR, after exercise, in comparison to the value before the exercise, was also observed. Similarly, in test II a statistically significant increase in blood systolic pressure and HR after the exercise was observed, in comparison to the pre-training value. In test II a statistically significant decrease in blood systolic pressure was found in comparison to test I. In test II a statistically insignificant improvement in physical performance (increase in number of performed rotations) and significant mental agility as well as skills and abilities (task simulation) were observed in comparison to test I (Tables 2, 3). In test II, in the assessment of synthetic (operational) memory, an improvement in errors was shown compared to test I (Figure 2).

**Table 2:** Physiological and psychomotor parameters results in cadets (*n* = 20).

Parameters	Test I M ± SD	95% CI	Test II M ± SD	95% CI	Cohen‘s d test	*F*	*p*
Systolic pressure before the exercise (mmHg)	141.1 ± 15.2	133.9; 148.2	133 0.6 ± 13.11	127.5; 139.7	0.45	2.76	0.09
Diastolic pressure before the exercise (mmHg)	89.7 ± 9.91	85.1; 94.3	90.7 ± 10.16	85.9; 95.4	0.08	0.08	0.76
Systolic pressure after the exercise (mmHg)	154.9** ±14.53	148.0; 161.7	143.7* ±19.63	134.5; 152.9	0.55	4.16	<0.05
Diastolic pressure after the exercise (mmHg)	93.6 ± 15.37	86.4; 100.7	91.7 ± 11.75	86.1; 97.2	0.10	0.19	0.66
Heart rate (HR) before the exercise (bpm)	84.8 ± 15.96	77.3; 92.3	84.0 ± 13.27	77.8; 90.2	0.04	0.03	0.86
Heart rate (HR) after the exercise (bpm)	125.25** ±24.38	113,8; 136,6	123.65** ±17.92	115.2; 132.0	0.05	0.05	0.81
Execution of rotations forward on looping (number)	49.45 ± 10.5	44.5; 54.3	54.1 ± 11.74	48.6; 59.5	0.34	1.74	0.19
Percentage completion of tasks during doing exercises on a looping (%)	73.7 ± 14.84	66.8; 80.6	87.25 ± 10.85	82.1; 92.3	0.81	10.77	<0.01

M ± SD – mean, standard deviation; CI – confidence interval; *p*-level of significance.

* *p* < 0.05 - statistically significant difference in comparison to the pre-exercise value.

** *p* < 0.001 - statistically significant difference in comparison to the pre-exercise value.

**Table 3:** Percentage distribution of the results in test I and II.

Tasks performance percentage	Test I *n* = 20	Test II *n* = 20
33	1	-
50	1	-
66	7	2
83	10	11
100	1	7

The rest of the parameters showed no changes. After cadets had 6 months of practice in the air, the effect size (d Cohen) value calculated. For the execution of forward rotation on looping, it was small, and for the percentage of completion of tasks during the exercises, it was large (Table 2).

**Figure 3:**
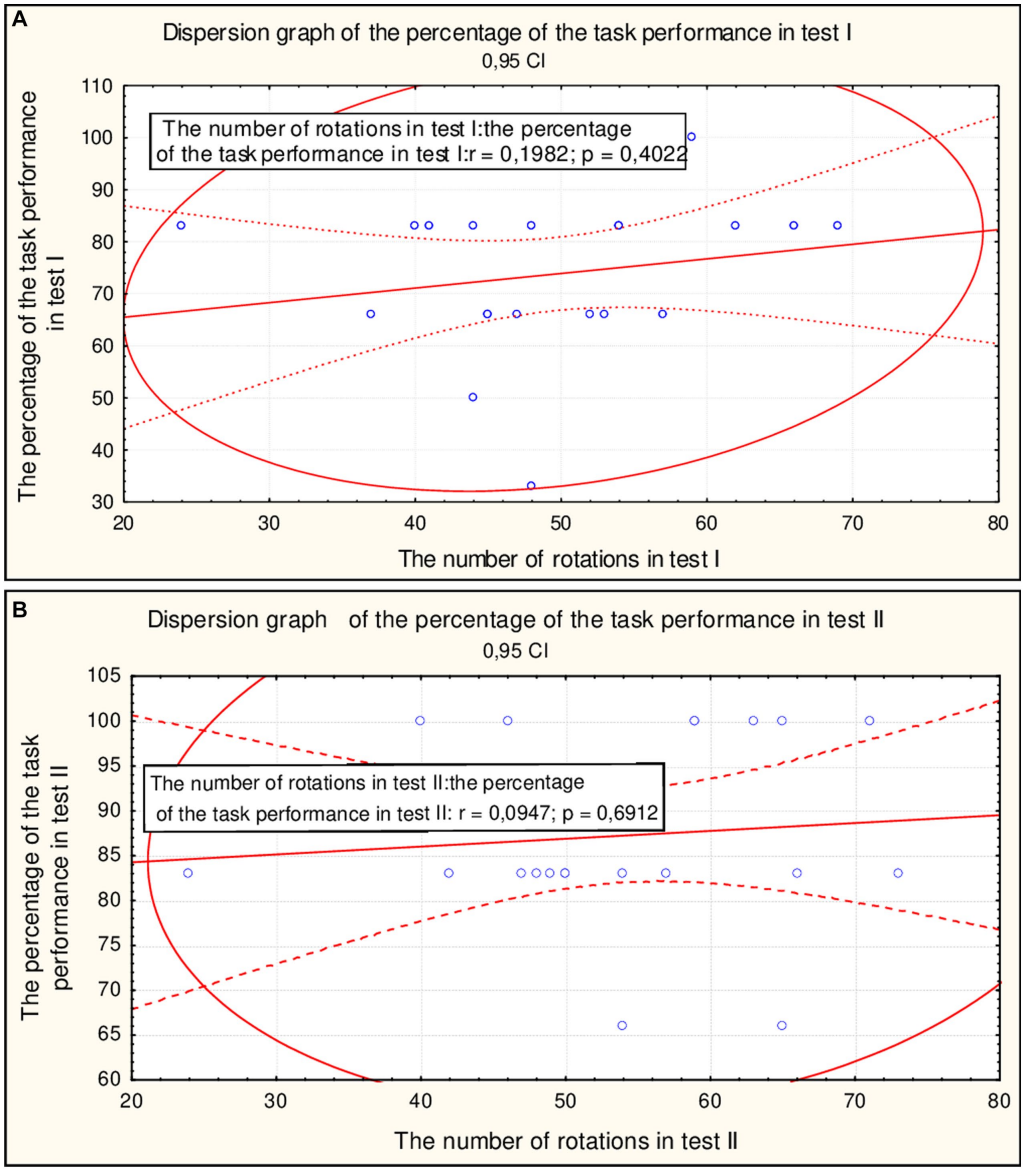
The correlation between the percentage of performed tasks’ and a number of rotation in the test I **(A)** and II (**B**; *n* = 20).

In test II, a lower correlation was found between the number of rotations and the percentage of task completion compared to test I (Figure 3).

## Discussion

Based on the conducted investigation, the authors assumed that the exploited diagnostic-training instrument will be of high diagnostic value and will make it possible to determine the level of cognitive performance depending on the intensity of looping exercises. The performance of the task in extreme conditions of the pilot’s working environment determines high physical and mental fitness. The results of the study showed that the physical effort in Test I and II was performed in the aerobic metabolic zone, as indicated by the results of the heart rate (HR) measurement. Looping exercises overburdened the body due to positive +Gz (head-leg direction) and negative-Gz (leg-head direction) overloads. The exercises on the looping are connected with a huge impact on the respective fields of arteries of the cardiovascular system and the body’s central nervous system. During these exercises, there is a short-term increase in high blood pressure and HR. Such reactions were also observed by Kłossowski (1994). The observed higher values of physiological parameters than the resting values in cadets before Tests I and II were probably caused by the emotions linked to performing a specific attempt. During the test, the student did not see a point of reference in space for their body (no view of the environment due to goggles covering the eyes). It was found that the somatic features of cadets between Tests I and II did not change significantly (Table 1). The applied maximum performance exercise (forward rotations on the looping) in the experiment combined with a simultaneous simulation of stimuli (tasks) appearing in the central field of vision caused complex sensory reactions in pilot cadets. The instructed response of the person being examined during the dynamic exercises while looping is to receive accurate information, processing it, and give a correct answer. The military pilot frequently encounters such situations during a flight. The execution of rotations was a constant movement stimulus, studied and adapted to the requirements of the military pilot’s working environment. The rotations were performed in accordance with the structural scope of the Schmidt motor program (Schmidt, 1976, 1991). It needs to be stressed that the transmitted stimuli (tasks) were displayed unexpectedly within 128 s during the execution of the rotations (the waiting time for displaying a task was unknown to the student). Detecting a stimulus (task), a proper reaction (answer), and speed of information processing, measured in time, forms the basis for an effective test execution by pilot cadets. The factor determining the effectiveness of sensory processes was the compatibility of the stimulus (task) and reaction (answer) during a physical effort (rotations). It was found that the greater the compatibility of a stimulus and a response, the shorter the answer time (Eimer, 1995; Masaki et al., 2000; Worringham and Kerr, 2000). The disturbance between a stimulus and a response could have led to a delay in processing the information with a simultaneous reduction in the number of executed rotations and committing an error in an answer. It was found that the efficiency of sensorimotor processes is the basis of human motor functioning with regard to the motor response not only of bodily components but also of the whole body (Osiński, 1990). The observations in this study demonstrated a relationship between a complex reaction (answer to questions) in the central field of vision and the intensity of effort (number of forward rotations). The intense physical effort resulted in a prolonged reaction time (answer to questions), which could have caused disturbances in the central nervous system due to decreased cerebral perfusion. The optimum number of rotations affected the response time (answer to questions), which, at the same time, was the borderline between an increase and a decrease in the number of rotations, during which the student was answering all questions correctly. This borderline point is the maximum level of psychomotor capability under extreme exercise conditions. In Test I, a positive correlation r = 0.19 was observed in all examined cadets between the number of performed rotations and the percentage execution of the tasks. This means that among the respondents, a higher level of the sensory response (answer to questions) and a lower level of the motor response (number of forward rotations) were found, while in others an opposite tendency was observed. This is due to the difference in the level of integration of neurosensory network connections with the motor ones. An intense physical effort and a delayed response time may cause a delay in the processing of information (Sanders, 1990) and a delay in answering, and, consequently, a reduction in the percentage of the sensory task execution (number of committed errors). This fact is confirmed by the studies of other authors (Yagi et al., 1999; Tomporowski, 2003; Kamijo et al. 2004; Ando et al., 2005; Thomson et al., 2009). Previous research (Prokopczyk and Wochyński, 2022a,b) shows that after the SAGI special training process, the percentage of task completion was statistically significantly higher than before the training process. It should be emphasized that in this study, the difference in the percentage of task completion before and after flight practice was also statistically significant, and their values were higher than in the special training process. The difference in the results of looping rotations before and after the training process was statistically insignificant (Prokopczyk and Wochyński, 2022a,b). Similarly, in this study, there was a non-significant difference in the values before and after the period of flying practice in looping rotations. It was shown that their values were higher than in the special training process. In this study, with a higher value of rotations performance on looping, a higher percentage of task completion was found in relation to the same variables in the training process on SAGI. It follows from this that the cadets after aviation practice had a higher degree of psychomotor skills. This fact is confirmed by the lower correlation between the percentage of task completion and the number of rotations performed on looping in this study in relation to the study of the special training process. The justification for a higher psychomotor level in cadets after air practice in relation to cadets in the training process at SAGI may result from their psychomotor predispositions or adaptation to the pilot’s work environment. It may also be a cumulative effect, which consisted of an earlier period of special preparation for flights on SAGIimmediately followed by the start of the aviation practice stage. In Test I, a synthetic (operational) memory test was performed after the looping exercises. The synthetic memory was disturbed in the examined students as a result of ongoing fatigue and stress resulting from time deficit activities, and greater concentration in responding to stimuli (questions). This fact is confirmed by some cadets, since on completion of the attempt, they did not remember the order of the performed tasks during the test. A large percentage discrepancy of tasks and a fewer number of rotations could have been caused by poor integration of the sensory-motor system. The findings of Test II revealed that the level of physical and mental efficiency depends on the course of the adaptation and integration processes of the two systems: the motor and the sensory one. In the timing criteria of executing the above-mentioned tasks, the level of physical and mental fitness may be the cause of the occurrence of feedback between these systems. This was evidenced by a decreasing correlation between the percentage execution of tasks and the number of completed rotations in Test II, as compared to Test I. In Test II (after half a year of flying practice in the air) better synthetic (operational) memory was observed, which is probably a result of increased sensory-motor activity and concentration of attention trained when flying. The test used in the study is a special exercise for pilots and may have triggered specific physiological reactions (lowered HR), indicating whether the pilot is adapted to physical effort in extreme conditions. A six-month practice period combined with physical education may have resulted in the inclusion of adaptive mechanisms, in which proteins and hormones could have played a crucial role. The previous author’s papers (Wochyński and Sobiech, 2014, 2015, 2017) demonstrated that the exercises on Special Aviation Gymnastic Instruments (SAGI; looping, gyroscope, aero wheel) lead to the adaptation to physical effort within 70 days, as a result of a special exercise programme for cadet pilots (Wochyński et al., 2010). It was found that dopamine in plasma, at the end of the preparation period, after the training unit, was high than before the training period (Wochyński and Sobiech, 2017). Such a concentration of dopamine, at the end of the training period, may indicate a fatigue delay and a positive regulation of the motor and limbic system. This was confirmed by studies in which dopamine plays an important role in cognitive processes and attention mechanisms (Nieoullon, 2002). The intensity of the looping exercise during the test could have affected the stimulation of the central nervous system. This type of exercise undoubtedly affects the vestibular organ, which undergoes habituation after prolonged exposure (Wochyński et al., 2020b). After a 6-month practice period (Test II), cadets showed a decrease in HR and diastolic pressure. Moreover, the significance of the difference was noted only in systolic blood pressure in relation to resting values and to Test I after the effort. The drop in blood pressure should be associated with smaller stress during Test II. It was observed that along with a decrease in HR, systolic and diastolic pressure, there was a percentage increase in tasks performed on the looping in Test II, which denotes a decrease in errors made by the cadets in Test II. An insignificant increase in the number of rotations in Test II shows an increase in the sensitivity of the neurosensory system (motor adjustment). The percentage enhancement in the task execution in Test II presumably results from improved visual and motor perception related to flying experience. The practical implementation of tasks in the air for half a year possibly resulted in adaptation to the occurring stress or its reduction due to mastering the operational activities in real conditions.

The authors wanted to emphasize that these studies (pilot) will contribute to a future experiment, with the possibility of using greater diagnostic accuracy and a larger number of people including the control group (according to the studied indicator for the entire population) for future candidates for military aviation. They will also be helpful in conducting training for cadets and instructors dealing with these issues in practice. The research will also be the basis for the use of special mental training for pilots, as in athletes (Piepiora et al., 2022).

## Conclusion

Summarizing the results of the study, it was found that practical pilotage training for pilot cadets contributed to the improvement of physical fitness and the efficiency of cognitive processes. A six-month practice, in the air, of pilot cadets and conducted physical education classes contributed to a percentage increase in task execution as well as in the number of rotations in Test II. The calculation of the Cohen effect size value shows that the practice period had a large impact on the percentage increase in the completion of tasks during exercises and a small effect on the increase in the number of rotations. The borderline point in maximum psychomotor abilities of the cadets was higher in Test II compared to Test I, together with a decreasing correlation between the percentage execution of tasks and making the rotations. The study has proved that the applied diagnostic and training instrument has a high diagnostic value in the assessment of the course of cognitive processes under conditions of a dynamic change of a body position in space. The device makes it possible to diagnose the level of pilots’ functioning in extreme conditions of the pilot’s working environment.

## Data availability statement

The original contributions presented in the study are included in the article/supplementary material, further inquiries can be directed to the corresponding author.

## Ethics statement

The authors achieved permission from the appropriate ethical commission to perform the study (decision No. 03A/2009, 08.07.2009, Ethical Commission on biomedical research studies at the Military Institute of Aviation Medicine in Warsaw). The study was conducted considering the guidelines of the Declaration of Helsinki.

## Author contributions

ZW contributed to the conception and design of the study and wrote the first draft of the manuscript. JS-R and ZK organized the database. ZW and ZK performed the statistical analysis. JS-R wrote sections of the manuscript. All authors contributed to the article and approved the submitted version.

## Funding

The research study was financed by the National Centre for Research and Development (No. O R/00 001706).

## Conflict of interest

The authors declare that the research was conducted in the absence of any commercial or financial relationships that could be construed as a potential conflict of interest.

## Publisher’s note

All claims expressed in this article are solely those of the authors and do not necessarily represent those of their affiliated organizations, or those of the publisher, the editors and the reviewers. Any product that may be evaluated in this article, or claim that may be made by its manufacturer, is not guaranteed or endorsed by the publisher.
